# Plant diversity effects on grassland productivity are robust to both nutrient enrichment and drought

**DOI:** 10.1098/rstb.2015.0277

**Published:** 2016-05-19

**Authors:** Dylan Craven, Forest Isbell, Pete Manning, John Connolly, Helge Bruelheide, Anne Ebeling, Christiane Roscher, Jasper van Ruijven, Alexandra Weigelt, Brian Wilsey, Carl Beierkuhnlein, Enrica de Luca, John N. Griffin, Yann Hautier, Andy Hector, Anke Jentsch, Jürgen Kreyling, Vojtech Lanta, Michel Loreau, Sebastian T. Meyer, Akira S. Mori, Shahid Naeem, Cecilia Palmborg, H. Wayne Polley, Peter B. Reich, Bernhard Schmid, Alrun Siebenkäs, Eric Seabloom, Madhav P. Thakur, David Tilman, Anja Vogel, Nico Eisenhauer

**Affiliations:** 1 German Centre for Integrative Biodiversity Research (iDiv), Halle-Jena-Leipzig, Deutscher Platz 5e, 04103 Leipzig, Germany; 2 Institute of Biology, Leipzig University, Johannisallee 21, 04103 Leipzig, Germany; 3 Department of Ecology, Evolution and Behavior, University of Minnesota Twin Cities, Saint Paul, MN 55108, USA; 4 Institute for Plant Sciences, University of Bern, 3013 Bern, Switzerland; 5 Biodiversity and Climate Research Centre, Senckenberg, Senckenberganlage 25, Frankfurt am Main, 60325, Germany; 6 Ecological and Environmental Modelling Group, School of Mathematics and Statistics, University College Dublin, Dublin 4, Republic of Ireland; 7 Institute of Biology/Geobotany and Botanical Garden, Martin Luther University Halle-Wittenberg, Halle, Germany; 8 Institute of Ecology, Friedrich Schiller University Jena, Dornburger Strasse 159, 07743 Jena, Germany; 9 Department of Physiological Diversity, Helmholtz Centre for Environmental Research, Leipzig, Germany; 10 Plant Ecology and Nature Conservation Group, Wageningen University, PO Box 47, 6700 AA Wageningen, The Netherlands; 11 Department of Ecology, Evolution and Organismal Biology, Iowa State University, Ames, IA 50011, USA; 12 Department of Biogeography, BayCEER, University of Bayreuth, 95440 Bayreuth, Germany; 13 Institute of Evolutionary Biology and Environmental Studies, University of Zurich, 8057 Zurich, Switzerland; 14 Department of Biosciences, College of Science, Swansea University, Swansea, Wales, UK; 15 Ecology and Biodiversity Group, Department of Biology, Utrecht University, Padualaan 8, 3584 Utrecht, The Netherlands; 16 Department of Plant Sciences, University of Oxford, Oxford, OX1 3RB, UK; 17 Department of Disturbance Ecology, BayCEER, University of Bayreuth, 95440 Bayreuth, Germany; 18 Institute of Botany and Landscape Ecology, Ernst-Moritz-Arndt University Greifswald, 17487 Greifswald, Germany; 19 Department of Botany, Faculty of Sciences, University of South Bohemia, Branisovska 31, 37005 Ceske Budejovice, Czech Republic; 20 Centre for Biodiversity Theory and Modelling, Theoretical and Experimental Ecology Station, Centre National de la Recherche Scientifique and Paul Sabatier University, 09200 Moulis, France; 21 Department of Ecology and Ecosystem Management, School of Life Sciences, Weihenstephan, Technische Universität München, 85354 Freising, Germany; 22 Graduate School of Environment and Information Sciences, Yokohama National University, 79-7 Tokiwadai, Hodogaya, Yokohama, Kanagawa, 240-8501, Japan; 23 Department of Ecology, Evolution, and Environmental Biology, Columbia University, New York, NY 10027, USA; 24 Department of Agricultural Research for Northern Sweden, Swedish University of Agricultural Sciences, 90183 Umea, Sweden; 25 USDA-ARS Grassland, Soil and Water Research Laboratory, Temple, TX 76502, USA; 26 Department of Forest Resources, University of Minnesota, 1530 North Cleveland Avenue, St. Paul, MN 55108, USA; 27 Hawkesbury Institute for the Environment, Western Sydney University, Penrith, New South Wales 2751, Australia; 28 Department of Community Ecology, Helmholtz Centre for Environmental Research UFZ, Theodor-Lieser Strasse 4, 06120 Halle, Germany

**Keywords:** plant diversity, global change drivers, resource amendment, resource reduction, soil nutrients, drought

## Abstract

Global change drivers are rapidly altering resource availability and biodiversity. While there is consensus that greater biodiversity increases the functioning of ecosystems, the extent to which biodiversity buffers ecosystem productivity in response to changes in resource availability remains unclear. We use data from 16 grassland experiments across North America and Europe that manipulated plant species richness and one of two essential resources—soil nutrients or water—to assess the direction and strength of the interaction between plant diversity and resource alteration on above-ground productivity and net biodiversity, complementarity, and selection effects. Despite strong increases in productivity with nutrient addition and decreases in productivity with drought, we found that resource alterations did not alter biodiversity–ecosystem functioning relationships. Our results suggest that these relationships are largely determined by increases in complementarity effects along plant species richness gradients. Although nutrient addition reduced complementarity effects at high diversity, this appears to be due to high biomass in monocultures under nutrient enrichment. Our results indicate that diversity and the complementarity of species are important regulators of grassland ecosystem productivity, regardless of changes in other drivers of ecosystem function.

## 1. Introduction

Anthropogenically driven environmental change presently affects a considerable proportion of Earth's ecosystems [1] and is rapidly altering their capacity to provide the many ecosystem functions and services needed by human societies [2,3]. Global change drivers significantly impact on ecosystem functioning and biodiversity [4], which also plays a vital role in controlling ecosystem functioning [3,5]. While it is well established that increased biodiversity [6] and resource availability [7] enhance productivity, particularly in grasslands, the effect of the interaction between these factors is less well understood [4,8]. Furthermore, there is evidence to suggest that interactions between resource availability and biodiversity could be positive [9], negative [10] or non-significant [4,11,12].

High biodiversity could enhance productivity responses to increases in resource availability, e.g. increased precipitation or nutrient enrichment, through a number of mechanisms [13]. These include the greater likelihood of responsive species, e.g. nitrophilous grasses, being present and dominating diverse mixtures (selection effects) and increased resource-use efficiency for nitrogen with diversity, even in the absence of legumes [14]. This latter mechanism could lead to a more efficient exploitation of additional resources [9]. However, resource amendment may also diminish the positive effects of biodiversity. For example, where nitrogen (N) is added, legumes may decline in abundance and reduce their rates of N fixation, thus reducing their effects on resource supply [15,16]. Complementarity effects operating via nutrient-based niche differentiation, e.g. differing nutrient foraging strategies [17], could also be lost if resource limitation shifts to another resource for which complementarity effects are weaker, e.g. other soil nutrients [18], light [19], CO_2_ [20] or water [21]. Furthermore, under ambient conditions the availability of growth-limiting resources changes along plant diversity gradients. In three long-term grassland diversity experiments, N mineralization rates increased with plant species diversity [22–25], potentially lowering nutrient limitation and leading to a weaker response to nutrient addition in high-diversity communities.

Where there is a reduction in resource availability, biodiversity can buffer productivity declines via a number of mechanisms. These include the greater likelihood of tolerant species being present (insurance effects of biodiversity; e.g. [26]), which maintain productivity in periods of drought or nutrient limitation [27]. Furthermore, high-diversity communities may be more likely to contain species that are able to access scarce resources during periods of stress, e.g. by accessing water from deeper soil layers [28] or nutrients from different depths in the rhizosphere [29]. While such mechanisms might buffer decreases of productivity in high-diversity communities on a proportional scale, as predicted by biodiversity–stability theory [30] and confirmed in experimental studies [11,31], more diverse communities might suffer greater losses in productivity on an absolute scale, because they have more biomass to lose [10].

Given this wide range of possible interactions between biodiversity and resource availability on productivity, we examined the net balance of these mechanisms by measuring the strength and direction of the interaction between plant species richness and resource availability. This was achieved by performing a meta-level analysis, which is distinct from meta-analysis in that we used raw data and did not calculate effect sizes [32,33]. This was done using data from 16 experimental grassland studies, where plant species richness and resource availability were orthogonally manipulated. In all cases, resource reduction took the form of water reduction, i.e. drought, while resource amendment was nutrient addition. Secondly, we quantified net biodiversity, complementarity and selection effects [34] within treatments to test the extent to which diversity effects were altered by plant species richness, resource availability and their interaction.

## 2. Material and methods

### (a) Data collection and preparation

We created our database by consulting recently published meta-analyses on biodiversity–ecosystem function relationships [4,35,36]. We identified three additional studies (A. Hector, D. Deutschman, S. Levin & S. Pacala, unpublished; C.K.M. Palmborg, unpublished; C. Rosher & A. Siebenkäs, unpublished) that met our selection criteria. These were grassland experiments across Europe and North America that crossed a sown (or planted) plant species richness gradient with global-change drivers that increased or reduced resource availability. Given the low number of experiments that fully crossed sown plant diversity with other global-change drivers, such as CO_2_ enrichment or temperature, we further narrowed our selection criteria to only include studies that increased resource availability by adding nutrients or decreased resource availability by reducing water availability, thus ensuring comparability of studies. We obtained datasets from 16 experiments (6 drought, 10 nutrient addition; electronic supplementary material, appendix 1 and table S1). Hector *et al*. unpublished was treated as two independent studies because sown plant species richness was independently crossed with nutrient addition and drought treatments. In the selected experiments, nutrients were added once per year as NPK fertilizer or ammonium nitrate (NH_4_NO_3_) and water availability was generally reduced using rain-out shelters (electronic supplementary material, table S1).

In total, the nutrient addition dataset comprises observations from 1199 plots (*n =* 4032), and the drought dataset consists of 788 plots (*n* = 2150). All observations are of above-ground plant productivity (g m^−2^) for each plot in each year of each experiment (electronic supplementary material, table S1 and figures S1 and S2). For experiments that increased nutrient availability, we used peak biomass (a proxy for ANPP), while for experiments that reduced water availability, we used post-drought biomass harvested immediately (usually within one week) following the termination of experimental drought treatments.

We calculated net biodiversity, complementarity and selection effects for the studies that recorded species level biomass (six nutrient addition studies and three drought studies; electronic supplementary material, table S1), following Loreau & Hector [34]. This subset of studies exhibited qualitatively similar above-ground productivity responses to both plant species richness and drought or nutrient addition using the complete dataset (electronic supplementary material, table S2 and figure S3). As the experimental treatments were also expected to drive variation in above-ground productivity, we standardized net diversity, complementarity and selection effects by the mean monoculture biomass of the corresponding treatment to compare the strengths of these effects across treatments. The standardized net diversity effect is the difference between the observed and expected above-ground productivity of a mixture, i.e. 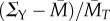, where *Σ*_Y_ is the observed yield, 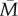 is the average above-ground productivity in a monoculture of each species in a plot, and 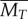 is the mean monoculture biomass of the treatment. The standardized complementarity effect reflects the extent to which species produce more (or less) biomass in a mixture than in a monoculture: 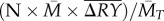, where N is the number of species and 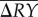 is 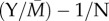. The standardized selection effect is 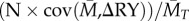 and describes whether species with high yields in monocultures also dominate in mixtures. Species with a mean monoculture biomass less than 2.5 g m^−2^ in a given year were excluded because relative yield can approach infinity when monoculture biomass values are close to zero [37].

### (b) Data analysis

For nutrient addition and drought studies, we fitted separate linear mixed-effects models that test for the effects of plant species richness, treatment and the interaction of the two on above-ground productivity. Plant species richness was the natural log of sown plant species richness and treatment was a binary variable (0, control; 1, treatment). Random effects were included for a study factor, interactions of study with plant species richness, treatment, time (year of experiment) and their interactions, and a plot (within study) term. We accounted for repeated measurements within plots by using a first-order autoregressive covariance structure, which fitted the data better than a compound symmetry covariance structure based on the Akaike information criterion. To test for the effects of plant species richness, treatment and the interaction of the two on net biodiversity, complementarity and selection effects, we fitted separate linear mixed-effects models for nutrient addition and drought studies. For nutrient addition studies, we used the same model structure described above; for drought studies, we simplified the aforementioned model due to model convergence issues. The simplified model contained the same fixed- and random-effects structure as the previous models, but did not include a temporal correlation covariance structure. Above-ground productivity was square-root transformed to meet model assumptions, which were checked by visually inspecting residual plots for homogeneity and quantile–quantile plots for normality. Models were fitted using the asreml function in the asreml package in R, and Wald tests and variance components were extracted using the test.asreml function in the pascal package (electronic supplementary material, appendix S2).

We performed sensitivity analyses to test whether results differed when excluding either low- (sown plant species richness = 1) or high-diversity communities (sown plant species richness greater than 20). To do so, we fitted the linear mixed-effects models to both subsets for each type of resource alteration as described above. For nutrient addition studies, we also tested the sensitivity of our results to the type of fertilizer used (NPK or NH_4_NO_3_) by fitting a linear mixed effects model where treatment changed to a variable with three levels (0, control; 1, NPK; 2, NH_4_NO_3_). Additionally, we assessed the robustness of our results to covariates: for nutrient addition experiments, the amount of nutrients added (N g m^−2^; electronic supplementary material, table S1), and for water reduction experiments, drought duration. The latter was used as a proxy for drought severity because of its significantly negative correlation with plant productivity across biomes [38]. Drought duration was strongly right-skewed and nonlinear because most studies excluded precipitation for fewer than 60 days, with the exception of two studies that did so for more than 130 days (electronic supplementary material, table S1). Thus, drought duration was treated as a categorical variable, where short droughts were those lasting less than 60 days and long droughts lasted longer than 60 days. We tested the influence of the above-mentioned covariates by fitting separate linear mixed effects models where a fixed effect for nutrient addition or drought duration and the corresponding interactions with plant species richness and treatment were added to the original models. All analyses were performed in R v. 3.2 [39].

## 3. Results

### (a) Above-ground productivity

**Figure 1.**
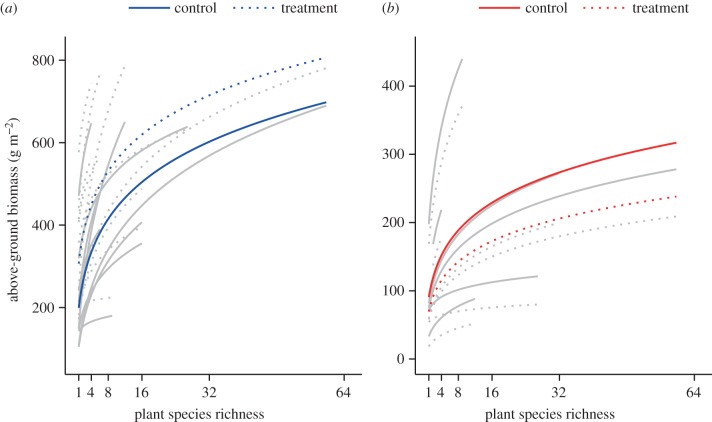
Plant species richness effects on productivity in response to (*a*) nutrient addition or (*b*) drought. Lines are mixed-effects model fits for each treatment within each study (light grey lines) or for each treatment across all studies (black lines). Solid lines refer to Control and dashed lines correspond to Treatment, where nutrient or water availability was experimentally manipulated. (Online version in colour.)

For both types of resource alteration, above-ground productivity varied significantly by plant species richness and treatment but there was no significant interaction between the two (table 1). Productivity increased significantly with plant species richness across both nutrient addition and drought studies (figure 1), yet exhibited contrasting responses to each treatment: productivity increased with nutrient addition and decreased with drought (on log-square root scale; table 2). While the effects of plant species richness and treatment were consistent among studies (see variance components, table 1), the interaction between plant species richness and resource alteration was highly variable among studies for both types of resource alteration (table 1). Among studies, productivity also varied strongly across experimental years for both types of resource alteration (table 1).

**Table 1.** Fixed effects and variance component estimates (standard error) for linear mixed-effects models of above-ground productivity response to plant species richness and nutrient addition or drought.

	nutrient addition	drought
fixed effects		
intercept	*F*_1,8.5_ = 141.1***	*F*_1,5.1_ = 46.93***
species richness	*F*_1,8.1_ = 29.29***	*F*_1,4.2_ = 17.13*
treatment	*F*_1,7.9_ = 18.13**	*F*_1,3.8_ = 19.08*
species richness × treatment	*F*_1,801.9_ = 1.29	*F*_1,309_ = 1.02
variance components		
study	14.69 (9.07)	9.66 (7.10)
study × species richness	2.11 (1.25)	0.94 (0.77)
study × treatment	1.97 (1.27)	0.08 (0.22)
study × species richness × treatment	0.000002 (0.00000006)^a^	0.000002 (0.00000008)^a^
study × time	4.20 (1.35)^a^	2.92 (1.21)^a^
Plot	13.86 (1.08)^a^	11.50 (0.99)^a^
temporal autocorrelation		
*ρ*AR(1)	0.10 (0.03)^a^	0.05 (0.05)

*p < 0.05; **p < 0.01; ***p < 0.001.

^a^The *z* ratio of the variance component is greater than 1.96. Above-ground productivity (square-root transformed for analysis) is the response variable for both models. Species richness is the number of sown plant species (natural-log transformed), Treatment is a factor where 0 is Control and 1 is Treatment (either nutrient addition or drought) and Time is the experimental year. Fixed effects were tested sequentially. Kenward–Roger approximations are given for denominator degrees of freedom.

**Table 2.** Fixed effects estimates and 95% CIs (on log-square root scale) for linear mixed effects models of above-ground productivity response to plant species richness and nutrient addition or drought.

	nutrient addition	drought
fixed effects		
intercept	14.12 (11.32, 16.91)	9.52 (6.77, 12.27)
species richness	3.01 (1.94, 4.08)	2.03 (1.10, 2.96)
treatment	3.39 (1.87, 4.91)	−1.18 (−2.13, −0.23)
species richness × treatment	−0.34 (−0.94, 0.25)	−0.29 (−0.86, 0.28)

Results from the sensitivity analyses, in which low- or high-diversity communities were excluded, were in agreement with the results derived from full datasets for both types of resource alteration (electronic supplementary material, tables S3 and S4). In all cases, productivity increased significantly with increasing plant species richness and varied significantly by treatment; but the interaction of plant species richness and treatment did not have significant effects on productivity. When accounting for the type of fertilizer (NPK or NH_4_NO_3_), we found similar results to the main analysis (i.e. figure 1*a* and table 1); above-ground productivity varied significantly with plant species richness (*F*_1, 8_ = 30.05, *p* = 0.0006) and treatment (*F*_2, 7.2_ = 14.74, *p* = 0.003) but there was no significant interaction between the two fixed effects (*p* > 0.10). Lastly, including the amount of nutrients added or drought duration as covariates did not explain significant amounts of variation in above-ground productivity (*p* > 0.10), nor did they alter the significant effects of either plant species richness or treatment on above-ground productivity.

### (b) Net diversity, complementarity and selection effects

**Figure 2.**
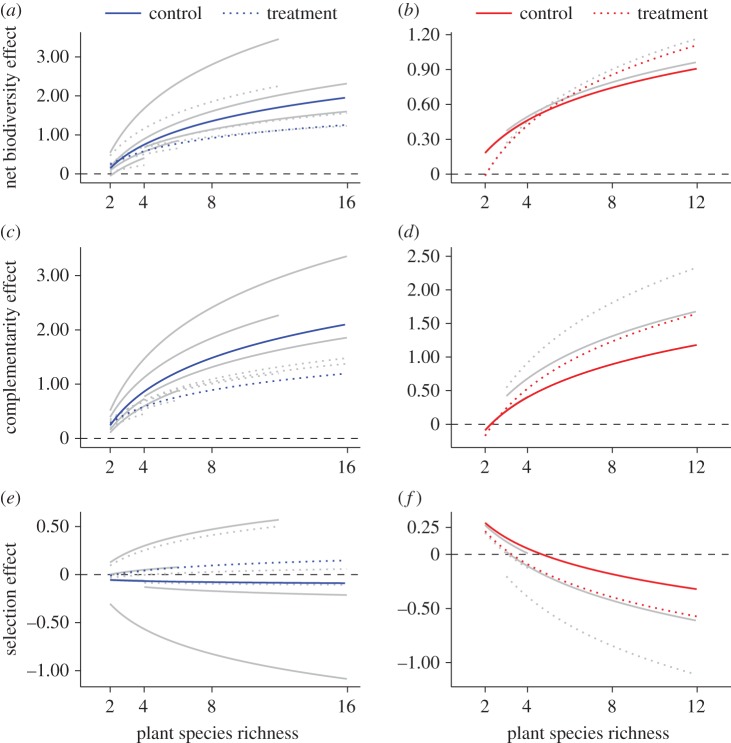
Plant species richness effects on net biodiversity, complementarity and selection effects in response to (*a*,*c*,*e*) nutrient addition and (*b*,*d*,*f*) drought. Lines are mixed-effects model fits for each treatment within each study (light grey lines) or for each treatment across all studies (black lines). Solid lines refer to Control and dashed lines correspond to Treatment, where nutrient or water availability was experimentally manipulated. Net biodiversity, complementarity and selection effects are dimensionless because they were standardized by the mean monoculture biomass of their corresponding treatment. (Online version in colour.)

With increasing plant species richness, net biodiversity and complementarity effects increased significantly in both nutrient addition and drought studies (figure 2; electronic supplementary material, table S5a and S5b). For nutrient addition studies, but not drought studies, the effect of plant species richness on net biodiversity and complementarity effects was significantly greater under control conditions than under nutrient enrichment (see interaction of plant species richness × treatment; electronic supplementary material, table S5a). Selection effects were not significantly reduced by plant species richness, treatment or their interaction in either nutrient addition or drought studies. The effects of plant species richness and the interaction between plant species richness and treatment on net biodiversity, complementarity and selection effects were consistent among nutrient addition studies (see variance components; electronic supplementary material, table S5a), as were those of plant species richness on net biodiversity, complementarity and selection effects among drought studies (electronic supplementary material, table S5b). However, the effects of resource alteration on net biodiversity, complementarity and selection effects among both nutrient addition and drought studies were highly variable.

## 4. Discussion

### (a) Effects of diversity on productivity

In our meta-level study, we found that plant diversity increased ecosystem productivity irrespective of nutrient or water availability. Our results suggest that nutrient enrichment and drought did not alter the strength or the direction of biodiversity–ecosystem function relationships in grassland plant communities despite significantly increasing and reducing productivity, respectively. This finding is consistent with previous studies that have found that positive biodiversity–ecosystem function relationships are robust to intensive management activities of grasslands, such as fertilization and mowing frequency [40,41], although function could still be impaired by biodiversity loss caused by fertilization and land use intensification [42–44]. These relationships appear to be driven primarily by increases in complementarity effects along plant species richness gradients, which were reduced by nutrient addition yet not affected by drought. Our results highlight the essential role of plant diversity in maintaining grassland productivity, which could be compromised by anthropogenic activities associated with biodiversity loss [45].

### (b) Effects of diversity and nutrient addition

Contrary to expectations that high-diversity communities may exploit additional resources to a greater extent than low-diversity communities [9], we found that changes in productivity caused by nutrient addition were constant along plant diversity gradients. Our results suggest that high-diversity communities failed to take advantage of nutrient enrichment due to its impact on complementarity, which was lower than in high-diversity communities under ambient conditions. The reduction of complementarity effects by nutrient enrichment probably reflects that above-ground productivity in monocultures was high under nutrient enrichment. This, as well as the non-significant reductions in selection effects under ambient conditions, contributed to the significant interaction of plant species richness and nutrient enrichment for net biodiversity effects, which was not observed for above-ground productivity. Nutrient enrichment probably reduces N inputs from legumes by decreasing N fixation and legume abundance [15,16,46], thus dampening the overall response of productivity to nutrient addition. Furthermore, our results suggest that diverse nutrient-uptake strategies that facilitate nutrient acquisition during different periods of the growing season [47] and from alternate sources and soil depths [28,29,48] probably become redundant when grassland communities are subjected to chronic nutrient enrichment. Thus, our results indicate that nutrient enrichment alters a crucial mechanism that maintains biodiversity. In the long term and in naturally assembled communities, the loss of this mechanism may result in the loss of species richness. Because biodiversity–productivity relationships are consistently positive, this in turn could offset some of the productivity gains made by increasing N availability [43,49,50].

### (c) Effects of diversity and drought

Irrespective of water availability, above-ground productivity increased significantly with plant diversity. This result is unexpected because drought is usually expected to strongly reduce above-ground productivity of high-diversity communities due to their greater overall biomass [27,51], as greater evapotranspiration increases vulnerability to drought [52], a weakening of complementarity effects, or the presence of species or plant functional groups that are sensitive to drought, such as legumes [10]. However, the non-significant effect of the interaction of plant species richness and drought on above-ground productivity suggests that such mechanisms were not operating. That drought did not affect net biodiversity or complementarity effects along plant species richness gradients supports the idea that diversity effects on above-ground productivity are robust to reductions in water availability. Repeated experimental droughts may select for specific, ‘conservative’ plant traits [53] that enhance drought tolerance [54,55] yet are more strongly correlated with survival than productivity [56]. In other words, while shifts in functional composition may occur in response to drought, they may not translate directly into changes in productivity [57]. Although grassland communities lose biomass during experimental droughts, they may conserve vital ecosystem functions by becoming progressively more tolerant to future droughts due to shifts in functional composition.

### (d) Variability across studies and underlying mechanisms

In this study, we found that the effect of the interaction between diversity and both types of resource alterations—nutrient enrichment and drought—on above-ground productivity was not significant. Non-significant interaction effects do not indicate that the hypothesized mechanisms were not operating (as indicated by our analysis of net biodiversity, complementarity and selection effects), but rather that they produced a response that was neutral across studies. The strong variation of site-level responses, as evidenced by the large variation in the effect of the interaction between plant diversity and treatment on above-ground productivity among studies, suggests that the relative balance of these mechanisms varied between studies, possibly due to differences in experimental design, site-level variation in resource limitations, and the adaptation of the local species pool to these limitations.

Lastly, environmental conditions, such as soil nutrient pools and precipitation, also varied over time and could have altered the realized magnitude of experimental treatments. Future research should therefore investigate the cause and importance of between-site variation in determining the interaction between resource availability and diversity, as it is possible that interactions could be stronger in certain ecosystems. This question may be best addressed using standardized methodology, e.g. by using global networks of standardized experiments that enable sources of across-site variation in environmental conditions to be quantified precisely [50,58,59].

## 5. Conclusion

The present results show that the positive effects of biodiversity on above-ground productivity are robust to resource alterations. This finding is consistent with other recent meta-analyses, which have found that the interaction between diversity and other drivers of ecosystem function is surprisingly weak in determining a range of ecosystem functions and properties, such as decomposition and soil microbial biomass carbon [4,12]. Importantly, our results revealed that one of the key mechanisms underlying biodiversity–ecosystem functioning relationships—complementarity—is not equally sensitive to nutrient enrichment and drought. Thus, the disruption of complementarity effects appears to be one of the key mechanisms that propagate changes in ecosystem dynamics in natural grasslands affected by chronic resource alterations such as N deposition [43,49,60]. By contrast, pulse disturbances such as drought may not alter the strength of diversity effects and, by extension, their ability to de-stabilize ecosystem functioning [11]. Together these results suggest that, while resource availability strongly impacts biodiversity, the relationship between biodiversity and ecosystem function is largely unaffected by resource availability, and, therefore, that biodiversity is a strong regulator of ecosystem function across a wide range of environmental conditions.

## Supplementary Material

Electronic supplementary materials

## Data accessibility

All sources included in this meta-level analysis are listed in the electronic supplementary material.
