# Supplementary material for: Plant diversity effects on grassland productivity are robust to both nutrient enrichment and drought
Source: Philos Trans R Soc Lond B Biol Sci. 2016 May 19;371(1694):20150277. doi: 10.1098/rstb.2015.0277 (PMC4843698; doi:10.1098/rstb.2015.0277)

**Electronic supplementary materials**

**Index**

**Appendix 1**. List of references of the published studies used in this meta-analysis……………….............**2**

**Appendix 2.** R code for fitting linear mixed-effects models……………………………………….………**3**

**Table S1.** List of studies……………………………………………………………………………….…...**5**

**Table S2**. Model summary for linear mixed-effects models of productivity responses using data from studies included in analysis of net biodiversity, complementarity, and selection effects .............................**6**

**Table S3.**Model summary for linear mixed-effects models of productivity responses to nutrient addition using data sets that excluded monocultures or high-diversity communities………………………………..**7**

**Table S4.** Model summary for linear mixed-effects models of productivity responses to drought using data sets that excluded monocultures or high-diversity communities………………………………….…..**8**

**Table S5a**. Model summary for linear mixed-effects models testing responses of net biodiversity (NBE), complementarity (CE), and selection (SE) effects to nutrient addition and plant species richness………...**9**

**Table S5b**. Model summary for linear mixed-effects models testing responses of net biodiversity (NBE), complementarity (CE), and selection (SE) effects to drought and plant species richness………………...**10**

**Figure S1**. Aboveground productivity along experimental plant species richness gradients for each experiment that manipulated nutrient availability……………...…………………………………………**11**

**Figure S2**. Aboveground productivity along experimental plant species richness gradients for each experiment that manipulated drought………...…………………….……….…………………………….**12**

**Figure S3.** Plant species richness effects on productivity in response to **a)** nutrient addition or **b)** drought using data from studies included in analysis of net biodiversity, complementarity, and selection effects………………………………………………………………………………………………….......**13**

**Appendix 1. List of references of the published studies used in this meta-analysis.**

[1] Isbell, F., Reich, P.B., Tilman, D., Hobbie, S.E., Polasky, S. & Binder, S. 2013 Nutrient enrichment, biodiversity loss, and consequent declines in ecosystem productivity. *Proceedings of the National Academy of Sciences* **110**, 11911-11916. (doi:10.1073/pnas.1310880110).

[2] Jentsch, A., Kreyling, J., Elmer, M., Gellesch, E., Glaser, B., Grant, K., Hein, R., Lara, M., Mirzae, H., Nadler, S.E., et al. 2011 Climate extremes initiate ecosystem-regulating functions while maintaining productivity. *Journal of Ecology* **99**, 689-702. (doi:10.1111/j.1365-2745.2011.01817.x).

[3] Fridley, J. 2002 Resource availability dominates and alters the relationship between species diversity and ecosystem productivity in experimental plant communities. *Oecologia* **132**, 271-277. (doi:10.1007/s00442-002-0965-x).

[4] Fridley, J.D. 2003 Diversity effects on production in different light and fertility environments: an experiment with communities of annual plants. *Journal of Ecology* **91**, 396-406. (doi:10.1046/j.1365-2745.2003.00775.x).

[5] Vogel, A., Scherer-Lorenzen, M. & Weigelt, A. 2012 Grassland Resistance and Resilience after Drought Depends on Management Intensity and Species Richness. *PLoS ONE* **7**, e36992. (doi:10.1371/journal.pone.0036992).

[6] Weigelt, A., Weisser, W.W., Buchmann, N. & Scherer-Lorenzen, M. 2009 Biodiversity for multifunctional grasslands: equal productivity in high-diversity low-input and low-diversity high-input systems. *Biogeosciences* **6**, 1695-1706. (doi:10.5194/bg-6-1695-2009).

[7] Lanta, V., Doležal, J., Zemková, L. & Lepš, J. 2012 Communities of different plant diversity respond similarly to drought stress: experimental evidence from field non-weeded and greenhouse conditions. *Naturwissenschaften* **99**, 473-482. (doi:10.1007/s00114-012-0922-4).

[8] Lanta, V. & Lepš, J. 2007 Effects of species and functional group richness on production in two fertility environments: an experiment with communities of perennial plants. *Acta Oecologica* **32**, 93-103. (doi:http://dx.doi.org/10.1016/j.actao.2007.03.007).

[9] Wilsey, B.J., Daneshgar, P.P. & Polley, H.W. 2011 Biodiversity, phenology and temporal niche differences between native- and novel exotic-dominated grasslands. *Perspectives in Plant Ecology, Evolution and Systematics* **13**, 265-276. (doi:http://dx.doi.org/10.1016/j.ppees.2011.07.002).

[10] Pfisterer, A.B. & Schmid, B. 2002 Diversity-dependent production can decrease the stability of ecosystem functioning. *Nature* **416**, 84-86.

[10] Rixen, C., Freppaz, M., Stoeckli, V., Huovinen, C., Huovinen, K. & Wipf, S. 2008 Altered Snow Density and Chemistry Change Soil Nitrogen Mineralization and Plant Growth. *Arctic, Antarctic, and Alpine Research* **40**, 568-575. (doi:10.1657/1523-0430(07-044)[RIXEN]2.0.CO;2).

[11] Roscher, C., Schmid, B., Kolle, O. & Schulze, E.D. 2016 Complementarity among four highly productive grassland species depends on resource availability. *Oecologia*, 1-12.

[12] Wacker, L., Baudois, O., Eichenberger-Glinz, S. & Schmid, B. 2009 Diversity effects in early- and mid-successional species pools along a nitrogen gradient. *Ecology* **90**, 637-648. (doi:10.2307/27651028)

**Appendix 2. R code for fitting linear mixed-effects models.**

install.packages('devtools')

library(devtools);install_github("pascal-niklaus/pascal/pascal")

require(asreml)

require(asremlPlus)

require(pascal)

require(MuMIn)

require(dplyr)

require(stringr)

require(reshape2)

###########

## data ####

###########

df<-read.table('data.csv',sep=",",header=T)

df$SppN<-as.numeric(df$SppN)

df<-arrange(df,Study,Plot,Time)

df$Time2 <- paste("y",df$Time,sep="")

df$Time2 <- factor(df$Time2)

#make balanced sparse data frame

dff <- expand.grid(UniquePlot=unique(df$UniquePlot),Time2=unique(df$Time2))

dff<- merge(dff,df[!duplicated(df[,which(names(df)%in%c("Study","UniquePlot"))]),

which(names(df)%in%c("Study","UniquePlot"))])

dff <-merge(dff,df[!duplicated(df[,which(names(df)%in%c("SppN","UniquePlot","Treatment",

"Biomass", "Time","Time2"))]),

which(names(df)%in%c("SppN","UniquePlot","Treatment","Biomass","Time","Time2"))],all.x=T)

dff <- dff[order(dff$UniquePlot,dff$Time),]

dff$Biomass[dff$Biomass==0] <- NA

dff <- arrange(dff,UniquePlot,Time2)

## possible data transformations for analysis

dff$lgBiomass<-log(dff$Biomass)

dff$sqrtBiomass<-sqrt(dff$Biomass)

dff$lgSppN<-log(dff$SppN)

dff$Treatment<-as.factor(dff$Treatment)

dff$Time<-as.numeric(dff$Time)

#######################

## Model ##############

#######################

#determine temporal covariance structure: compare cor(), AR(1)

#using AIC

#temporal covariance structure: AR(1)

m1<-asreml(fixed=sqrtBiomass~ lgSppN+ Treatment+lgSppN:Treatment,

random=~Study/(lgSppN*Treatment+Time2)+UniquePlot, rcov=~id(UniquePlot):ar1(Time2),na.method.X="include",keep.order=T,

control=asreml.control(maxiter=500),data=dff)

AIC1<- info.crit.asreml(m1)[2]

##evaluate model assumptions

plot(m1)

#extract Wald tests and variance components

test.asreml(m1)

#extract model coefficients

coefTable(m1)

**Table S1.** List of studies used for this meta-analysis that simultaneously manipulated plant species richness and resource availability. * Indicates studies used to calculate net diversity, complementarity, and selection effects.

| **Study** | **Location** | **Years** | **Duration (Years)** | **Species richness gradient** | **Experimental Treatments** | | |
| --- | --- | --- | --- | --- | --- | --- | --- |
|  |  |  |  |  | **Nutrient addition (N g m ^-2^)** | **Nutrients added** | **Mean drought days** |
| Isbell et al. 2013* | BioCON Experiment, Minnesota, USA | 1998 - 2011 | 14 | 1 - 16 | 4 | NH_4_NO_3_ |  |
| C. K. M. Palmborg, unpublished data * | Umea, Sweden | 2002 - 2003 | 2 | 1- 12 | 5 | NH_4_NO_3_ |  |
| A. Jentsch et al. 2011 | EVENT Experiment , Bayreuth, Germany | 2005 - 2010 | 6 | 2 - 4 |  |  | 37 |
| Fridley 2002, 2003* | North Carolina, USA | 2001 | 1 | 1 - 6 | 90 | N, P, K |  |
| Hector et al, unpublished data | Silwood Park, UK | 2000 - 2001 | 2 | 1 - 27 |  |  | 150 |
| Hector et al., unpublished data | Silwood Park, UK | 2000 - 2001 | 2 | 1 - 27 | 15 | NH_4_NO_3_ |  |
| A. Vogel et al. 2012 | Jena Experiment, Jena, Germany | 2009 - 2012 | 4 | 1 - 60 |  |  | 45.75 |
| A. Weigelt et al. 2009 | Jena Experiment, Jena, Germany | 2006 - 2009 | 4 | 1 - 60 | 10 | N, P, K |  |
| V. Lanta et al. 2012* | Benesov/Lipou, Czech Republic | 2003 - 2005 | 3 | 1 - 12 |  |  | 130.33 |
| V. Lanta and J. Leps 2007* | Benesov/Lipou, Czech Republic | 2003 - 2005 | 3 | 1 - 16 | 40 | N, P, K |  |
| B. Wilsey et al. 2011* | Texas, USA | 2008 - 2010 | 3 | 1 - 9 |  |  | 31 |
| A. B. Pfisterer and B. Schmid 2002* | Lupsingen, Switzerland | 1998 | 1 | 1 - 32 |  |  | 60 |
| C. Rixen et al. 2008 | Davos, Switzerland | 2000 – 2001 | 2 | 1 - 9 | 17 | NH_4_NO_3_ |  |
| C. Roscher, et al. 2016* | Jena, Germany | 2009 | 1 | 1 - 4 | 15 | N, P, K |  |
| Roscher & Siebenkäs, unpublished data | Bad Lauchstadt, Germany | 2013 | 1 | 1 - 4 | 12 | N, P, K |  |
| L. Wacker et al. 2009* | Zurich, Switzerland | 2002 | 1 | 1- 6 | 8, 16, 24 | N, Mg, S |  |

**Table S2**. Fixed effects tests and variance component estimates (standard error) for linear mixed-effects models of productivity responses to nutrient addition and drought using data from studies included in analysis of net biodiversity, complementarity, and selection effects.

|  | **Nutrient addition** | **Drought** |
| --- | --- | --- |
| **Fixed effects** |  |  |
| Intercept | F_1,4.8_ = 70.06*** | F_1,2.1_ = 10.19^ |
| Species richness | F_1,4.5_ = 31.38** | F_1,2_ = 11.72^ |
| Treatment | F_1,4.5_ = 16.07* | F_1,1.5_ = 13.32^ |
| Species richness *x* Treatment | F_1,540.5_ =1.74 | F_1,108.4_= 0.53 |
| **Variance components** |  |  |
| Study | 20.66 (15.84) | 22.68 (25.31) |
| Study *x* Species richness | 1.58 (1.29) | 0.88 (1.10) |
| Study *x* Treatment | 1.60 (1.38) | 0.07 (0.35) |
| Study *x* Species richness *x* Treatment | 0.000002 (0.00000007)  ^Ɨ^ | 0.000002 (0.0000001) ^Ɨ^ |
| Study *x* Time | 4.06 (1.49) ^Ɨ^ | 5.11 (3.64) |
| Plot | 16.47 (1.44) ^Ɨ^ | 13.63 (1.72) ^Ɨ^ |
| **Temporal autocorrelation** |  |  |
| ρ_AR(1)_ | 0.13 (0.03)  ^Ɨ^ | 0.05 (0.08) |

* P < 0.05, ** P < 0.01; *** P < 0.001, ^ P < 0.10, and ^Ɨ^ indicates that the z-ratio of the variance component is greater than 1.96. Biomass (square-root transformed for analysis) is the response variable for both models. Species richness is the number of sown plant species (natural-log transformed), Treatment is a factor where 0 is Control and 1 is Treatment (either nutrient addition or drought), and Time is the experimental year. Fixed effects were tested sequentially. Kenward-Roger approximations are given for denominator degrees of freedom.

**Table S3**. Fixed effects tests and variance component estimates (standard error) for linear mixed-effects models of productivity responses to nutrient addition using data sets that excluded monocultures or high-diversity communities.

|  |  | **Nutrient addition** | | |  |  |
| --- | --- | --- | --- | --- | --- | --- |
|  |  | No monocultures  (plant species richness: 2 – 60) | | No high diversity  (plant species richness: 1 – 16) | | |
| **Fixed effects** | |  |  | | |  |
|  | Intercept | F_1,8_ = 154.3*** | F_1,8.5_=141.8*** | | |  |
|  | Species richness | F_1,6.7_=43.81*** | F_1,8.2_=28.62*** | | |  |
|  | Treatment | F_1,7.8_=13.64** | F_1,7.9_=17.48** | | |  |
|  | Species richness *x* Treatment | F_1,468.3_=2.54 | F_1,785.3_=1.53 | | |  |
| **Variance components** | |  |  | | |  |
|  | Study | 11.33 (8.86) | 14.50 (9.05) | | |  |
|  | Study *x* Species richness | 1.59 (1.25) | 2.12 (1.27) | | |  |
|  | Study *x* Treatment | 2.24 (1.52) | 2.04 (1.31) | | |  |
|  | Study *x* Species richness *x* Treatment | 0.000002 (0.00000007)^Ɨ^ | 0.000002 (0.00000006) ^Ɨ^ | | |  |
|  | Study *x* Time | 6.10 (1.98) ^Ɨ^ | 4.34 (1.40) ^Ɨ^ | | |  |
|  | Plot | 8.42 (0.98) ^Ɨ^ | 14.21 (1.11) ^Ɨ^ | | |  |
| **Temporal autocorrelation** | |  |  | | |  |
|  | ρ_AR1_ | 0.04 (0.04) | 0.10 (0.03) ^Ɨ^ | | |  |

* P < 0.05, ** P < 0.01; *** P <0.001, ^ P < 0.10, and ^Ɨ^ indicates that the z-ratio of the variance component is greater than 1.96. Biomass (square-root transformed for analysis) is the response variable for both models. Species richness is the number of sown plant species (natural-log transformed), Treatment is a factor where 0 is Control and 1 is Treatment (i.e., ‘nutrient addition’), and Time is the experimental year. Fixed effects were tested sequentially. Kenward-Roger approximations are given for denominator degrees of freedom.

**Table S4**. Fixed effects tests and variance component estimates (standard error) for linear mixed-effects models of productivity responses to drought using data sets that excluded monocultures or high-diversity communities.

|  |  | **Drought** | | |  |  |
| --- | --- | --- | --- | --- | --- | --- |
|  |  | No monocultures  (plant species richness: 2 – 60) | | No high diversity  (plant species richness: 1 – 16) | | |
| **Fixed effects** | |  |  | | |  |
|  | Intercept | F_1,3.8_ =54.23** | F_1,5.1_=47.09*** | | |  |
|  | Species richness | F_1,4.1_=10.77* | F_1,4.2_=21.41** | | |  |
|  | Treatment | F_1,4.5_=9.62* | F_1,15.2_=18.85*** | | |  |
|  | Species richness *x* Treatment | F_1,410.3_=2.34 | F_1,9.4_=0.74 | | |  |
| **Variance components** | |  |  | | |  |
|  | Study | 4.43 (5.35) | 9.90 (7.23) | | |  |
|  | Study *x* Species richness | 3.10 (2.60) | 0.73 (0.67) | | |  |
|  | Study *x* Treatment | 0.44 (0.46) | 0.000005 (0.0000002) ^Ɨ^ | | |  |
|  | Study *x* Species richness *x* Treatment | 0.000001 (0.00000005)^Ɨ^ | 0.04 (0.11) | | |  |
|  | Study *x* Time | 3.17 (1.30) ^Ɨ^ | 2.96 (1.23) ^Ɨ^ | | |  |
|  | Plot | 5.88 (0.69) ^Ɨ^ | 11.83 (1.03) ^Ɨ^ | | |  |
| **Temporal autocorrelation** | |  |  | | |  |
|  | ρ_AR1_ | - 0.02 (0.05) | 0.05 (0.05) ^Ɨ^ | | |  |

* P < 0.05, ** P < 0.01; *** P <0.001, ^ P < 0.10, and ^Ɨ^ indicates that the z-ratio of the variance component is greater than 1.96. Biomass (square-root transformed for analysis) is the response variable for both models. Species richness is the number of sown plant species (natural-log transformed), Treatment is a factor where 0 is Control and 1 is Treatment (i.e. ‘drought’), and Time is the experimental year. Fixed effects were tested sequentially. Kenward-Roger approximations are given for denominator degrees of freedom.

**Table S5a**. Fixed effects tests and variance component estimates (standard error) for linear mixed-effects models testing responses of net biodiversity (NBE), complementarity (CE), and selection (SE) effects to nutrient addition and plant species richness.

|  | **NBE** | **CE** | **SE** |
| --- | --- | --- | --- |
| **Fixed effects** |  |  |  |
| Intercept | F_1,3.9_ = 0.46 | F_1,3.5_ = 1.54 | F_1,25.9_= 0.05 |
| Species richness | F_1,4.9_ = 14.53* | F_1,4.9_ = 23.45** | F_1,8.6_=0.07 |
| Treatment | F_1,16.1_ = 0.001 | F_1,16.7_ =0.28 | F_1,14.1_ = 0.25 |
| Species richness *x* Treatment | F_1,14.4_ =9.01** | F_1,16.1_= 5.09* | F_1,19.6_=0.24 |
| **Variance components** |  |  |  |
| Study | 0.06  (0.10) | 0.02  (0.12) | 0.0000004 (0.00000002) ^Ɨ^ |
| Study *x* Species richness | 0.14  (0.11) | 0.04  (0.06) | 0.02  (0.03) |
| Study *x* Treatment | 0.0000003  (0.00000001) ^Ɨ^ | 0.0000001 (0.000000005) ^Ɨ^ | 0.0000001 (0.00000001) ^Ɨ^ |
| Study *x* Species richness *x* Treatment | 0.01  (0.01) | 0.03  (0.03) | 0.02  (0.02) |
| Study *x* Time | 0.14  (0.05) ^Ɨ^ | 0.22  (0.08) ^Ɨ^ | 0.08  (0.03) ^Ɨ^ |
| Plot | 0.11  (0.02) ^Ɨ^ | 0.37  (0.06) ^Ɨ^ | 0.54  (0.06) ^Ɨ^ |
| **Temporal autocorrelation** |  |  |  |
| ρ_AR(1)_ | 0.04  (0.04) | -0.19  (0.04) ^Ɨ^ | -0.51  (0.03) ^Ɨ^ |

* P < 0.05, ** P < 0.01; *** P < 0.001, ^ P < 0.10, and ^Ɨ^ indicates that the z-ratio of the variance component is greater than 1.96. Species richness is the number of sown plant species (natural-log transformed), Treatment is a factor where 0 is Control and 1 is Treatment (i.e. ‘nutrient addition’), and Time is the experimental year. Fixed effects were tested sequentially. Kenward-Roger approximations are given for denominator degrees of freedom.

**Table S5b**. Fixed effects tests and variance component estimates (standard error) for linear mixed-effects models testing responses of net biodiversity (NBE), complementarity (CE), and selection (SE) effects to drought and plant species richness.

|  | **NBE** | **CE** | **SE** |
| --- | --- | --- | --- |
| **Fixed effects** |  |  |  |
| Intercept | F_1,2.3_ = 30.45* | F_1,9.2_ = 0.01 | F_1,10.1_ = 0.92 |
| Species richness | F_1,7.5_ = 25.49** | F_1,3.9_ = 9.20* | F_1,3.3_ = 3.42 |
| Treatment | F_1,604_ = 0.61 | F_1,1.5_ = 0.88 | F_1,2.1_ = 0.55 |
| Species richness *x* Treatment | F_1,604.1_ =1.61 | F_1,28.4_= 0.72 | F_1,13.6_= 0.14 |
| **Variance components** |  |  |  |
| Study | 0.0000001 (0.00000001)^Ɨ^ | 0.000008 (0.0000005) ^Ɨ^ | 0.0000002 (0.00000001) ^Ɨ^ |
| Study *x* Species richness | 0.002  (0.01) | 0.09  (0.12) | 0.05  (0.08) |
| Study *x* Treatment | 0.000002 (0.0000001)^Ɨ^ | 0.000008 (0.0000005) ^Ɨ^ | 0.000004 (0.0000002) ^Ɨ^ |
| Study *x* Species richness *x* Treatment | 0.0000001 (0.00000001)^Ɨ^ | 0.01  (0.03) | 0.01  (0.02) |
| Study *x* Time | 0.04  (0.04) | 0.03  (0.06) | 0.06  (0.06) |

* P < 0.05, ** P < 0.01; *** P < 0.001, ^ P < 0.10, and ^Ɨ^ ndicates that the z-ratio of the variance component is greater than 1.96. Species richness is the number of sown plant species (natural-log transformed), Treatment is a factor where 0 is Control and 1 is Treatment (i.e. ‘drought’), and Time is the experimental year. Fixed effects were tested sequentially. Kenward-Roger approximations are given for denominator degrees of freedom.

**Figure S1.** Aboveground productivity along experimental plant species richness gradients for experiment that manipulated nutrient availability.


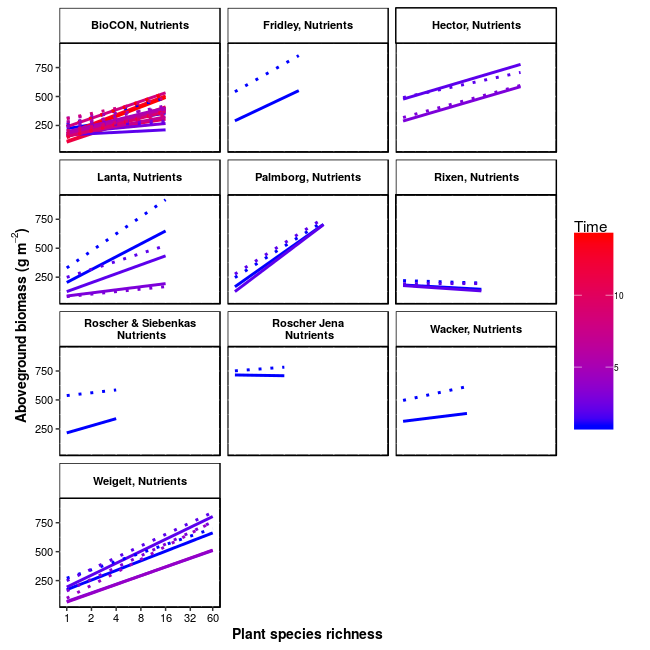


Lines are predicted lines from linear regression models. Solid lines refer to Control (no manipulation of nutrient availability) and dashed lines correspond to Treatment, where nutrient availability was experimentally manipulated, i.e., ‘nutrient addition’. Time is experimental years.

**Figure S2.** Aboveground productivity along experimental plant species richness gradients for each experiment that manipulated drought.


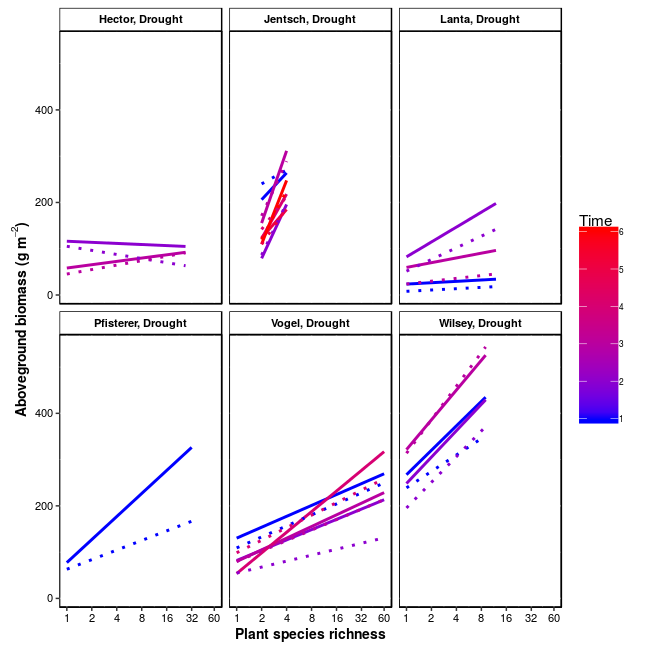


Lines are predicted lines from linear regression models. Solid lines refer to Control (no manipulation of nutrient availability) and dashed lines correspond to Treatment, i.e., where water availability was experimentally manipulated (‘drought’). Time is experimental years.

**Figure S3.** Plant species richness effects on productivity in response to **a)** nutrient addition or **b)** drought using data from studies included in analysis of net biodiversity, complementarity, and selection effects (see Table S2 for model fit information). Lines are mixed-effects model fits for each treatment within each study (gray lines) or for each treatment across all studies (blue = nutrient addition (6 studies), red = drought (3 studies)). Solid lines refer to Control and dashed lines correspond to Treatment, where nutrient or water availability was experimentally manipulated.


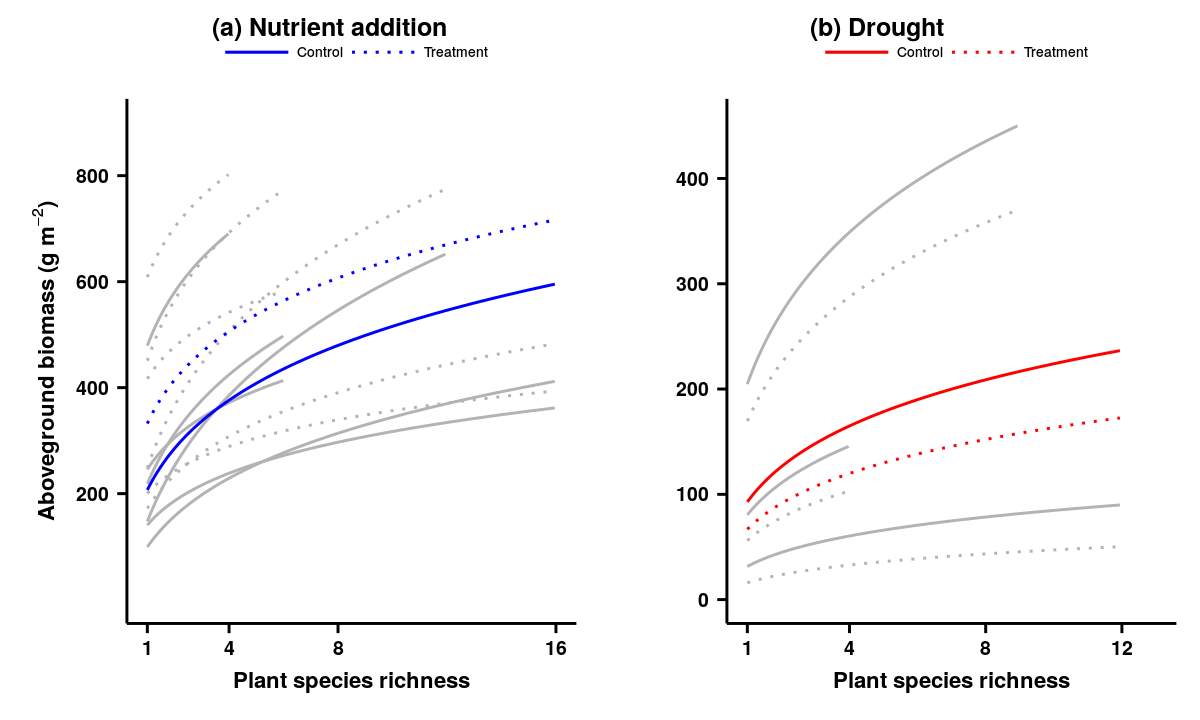

Supplement: Electronic supplementary materials [file rstb20150277supp1.docx]
